# Modeling binding specificities of transcription factor pairs with random forests

**DOI:** 10.1186/s12859-022-04734-7

**Published:** 2022-06-03

**Authors:** Anni A. Antikainen, Markus Heinonen, Harri Lähdesmäki

**Affiliations:** 1 Department of Computer Science, Aalto University, 02150 Espoo, Finland; 2 Folkhälsan Institute of Genetics, Folkhälsan Research Center, 00290 Helsinki, Finland; 3 Research Program for Clinical and Molecular Metabolism, Faculty of Medicine, University of Helsinki, Helsinki, 00290 Finland; 4 Helsinki Institute for Information Technology, Helsinki, Finland

**Keywords:** Transcription factor pair, Random forest, DNA binding site

## Abstract

**Background:**

Transcription factors (TFs) bind regulatory DNA regions with sequence specificity, form complexes and regulate gene expression. In cooperative TF-TF binding, two transcription factors bind onto a shared DNA binding site as a pair. Previous work has demonstrated pairwise TF-TF-DNA interactions with position weight matrices (PWMs), which may however not sufficiently take into account the complexity and flexibility of pairwise binding.

**Results:**

We propose two random forest (RF) methods for joint TF-TF binding site prediction: ComBind and JointRF. We train models with previously published large-scale CAP-SELEX DNA libraries, which comprise DNA sequences enriched for binding of a selected TF pair. JointRF builds a random forest with sub-sequences selected from CAP-SELEX DNA reads with previously proposed pairwise PWM. JointRF outperforms (area under receiver operating characteristics curve, AUROC, 0.75) the current state-of-the-art method i.e. orientation and spacing specific pairwise PWMs (AUROC 0.59). Thus, JointRF may be utilized to improve prediction accuracy for pre-determined binding preferences. However, pairwise TF binding is currently considered flexible; a pair may bind DNA with different orientations and amounts of dinucleotide gaps or overlap between the two motifs. Thus, we developed ComBind, which utilizes random forests by considering simultaneously multiple orientations and spacings of the two factors. Our approach outperforms (AUROC 0.78) PWMs, as well as JointRF (*p*<0.00195). ComBind provides an approach for predicting TF-TF binding sites without prior knowledge on pairwise binding preferences. However, more research is needed to assess ComBind eligibility for practical applications.

**Conclusions:**

Random forest is well suited for modeling pairwise TF-TF-DNA binding specificities, and ComBind provides an improvement to pairwise binding site prediction accuracy.

**Supplementary Information:**

The online version contains supplementary material available at 10.1186/s12859-022-04734-7.

## Background

Transcription factors (TF) bind enhancers and gene promoter regions in a sequence-specific manner to initiate or suppress target gene expression. Defining TF binding specificities, and hence the target genes, is the first step in understanding gene expression networks and thus e.g. cell differentiation patterns and responses to external stimuli [1]. However, understanding TF-DNA binding still remains a challenge. Transcription factor binding is probabilistic in nature and depends on DNA shape and methylation [2–5]. Moreover, TFs may exhibit multiple distinct binding specificities i.e. motifs [6, 7]. TFs cooperate with each other by facilitating each others binding, for instance through chromatin opening [8], and by forming DNA binding complexes [9]. In [10], Wunderlich et al. suggested TF cooperation to be of great importance especially in eukaryotes. Later on, Jolma et al. demonstrated that TF pairs can bind DNA with novel motifs, different configurations and clear spacing preferences [11]. To date, roughly 1600 TFs have been identified [1], thus, the amount of possible pairings is large. As the cooperativity of TFs has become increasingly clear, complex models to represent and predict pairwise TF binding are needed.

DNA binding sites are often experimentally searched in vitro—thus, minimizing the effects of chromatin state in motif discovery—with high-throughput methods such as protein binding microarrays and systematic evolution of ligands by exponential enrichment (SELEX) followed by sequencing (SELEX-seq) [1, 12]. Experimental methods have been further developed to enable binding specificity discovery of TF-TF complexes [11]. In [11], Jolma et al. studied TF-TF-DNA binding with a SELEX-seq based method; consecutive affinity purification evolution of ligands by exponential enrichment (CAP-SELEX), in which DNA sequences are purified for TF-TF binding and sequenced [7, 11, 12]. They studied 9,400 TF pairs and found that 315 of them exhibited pairwise DNA binding affinities; even across different TF classes [11]. Many TFs contain protein interaction domains with which they form complexes before binding DNA [9]. However, Jolma et al. suggested that TF pairs may bind DNA also with a manner facilitated by DNA, as TFs were bound on opposite strands or relatively far from each other [11]. CAP-SELEX data sets comprise hundreds of thousands of short DNA sequences known to entail a TF-TF binding site [11], thus, enabling the development of complex binding specificity models.

Transcription factor binding is probabilistic and the binding sites exhibit base position dependencies [2, 13]. Binding specificities are most commonly modeled with position weight matrices (PWMs), which describe probabilities to detect nucleic acids at each binding site position; sequence scores are attained by summing the logarithmic position specific scores. These matrices are derived from high-throughput data sets with motif discovery algorithms (e.g. MEME algorithm [14]). Position weight matrices are simple and intuitive, but have limitations. Nucleic acids are assumed to be independent of each other and thus the commonly occurring base correlations are ignored [13, 15]. Furthermore, base positions are independently normalized during PWM construction from position frequency matrices, which Ruan et al. suggested to be a significant source of inaccuracy [16]. In fact, binding specificities have been modeled more accurately for instance with hidden Markov models, k-mer models and neural network models [13, 15, 17, 18]. Position weight matrices may be especially unsuitable for TF complexes due to the assumption of a fixed binding configuration, since in [11], Jolma et al. suggested that TF pairs can bind DNA with multiple orientation and spacing preferences. Defining several PWMs for a single pair is an option to overcome multiple spacing preferences, but might not sufficiently explain the entire range of flexibility in pairwise binding. However, complex models for pairwise TF-TF binding are scarce. In [19], Hong et al. proposed a novel support vector machine (SVM) classifier for TF-TF binding site predictions, which was evaluated with 30 CAP-SELEX data sets each reduced to 800 DNA sequences. Despite the simplicity and evident limitations of PWMs, they still remain the most commonly used binding specificity description.

Random forest (RF) is an intriguing option for modeling TF-DNA binding specificities due to its capability to effectively model high-dimensional data with correlated variables following the grouping property of decision trees [20, 21]. Of note, decision trees are able to handle both categorical and numerical features. Decision trees are able to identify class specific clusters of correlated variables and give a small minimal depth to them [21], resulting in high classification accuracy for instances encompassing variables from these clusters. Importantly, variables (i.e. DNA base pairs) on TF-DNA binding site are usually correlated [15]. One of the most important benefits of RF is its ability to process large data sets, which currently is a requirement in genetics with all the novel high-throughput methodologies. In general, random forest has showcased good prediction accuracies in comparison to other machine learning tools in the field of life sciences with omics data [22]. Of note, RF has been exploited for modeling TF-DNA interactions with *in vivo* ChIP-seq data by Wang et al. [23] and Arkadani et al. [24]. Random forest, as an ensemble method, can achieve high model complexity and is able to process large genetic data sets obtained with high-throughput methods.

In this paper, we propose a novel random forest based pairwise binding specificity model, ComBind, trained with high-throughput CAP-SELEX sequence libraries from [11]. ComBind entail multiple TF-TF orientations and spacings in one binding prediction score. Thus, although ComBind has higher accuracy, it cannot distinguish individual binding preferences. Furthermore, we show that random forest can be combined with known pairwise PWMs to increase configuration specific prediction accuracy with a second RF based method: JointRF. ComBind outperforms PWMs, as well as JointRF, thus providing a notable improvement to pairwise TF-TF-DNA binding site prediction accuracy.

## Results

### Experimental setting

**Fig. 1:**
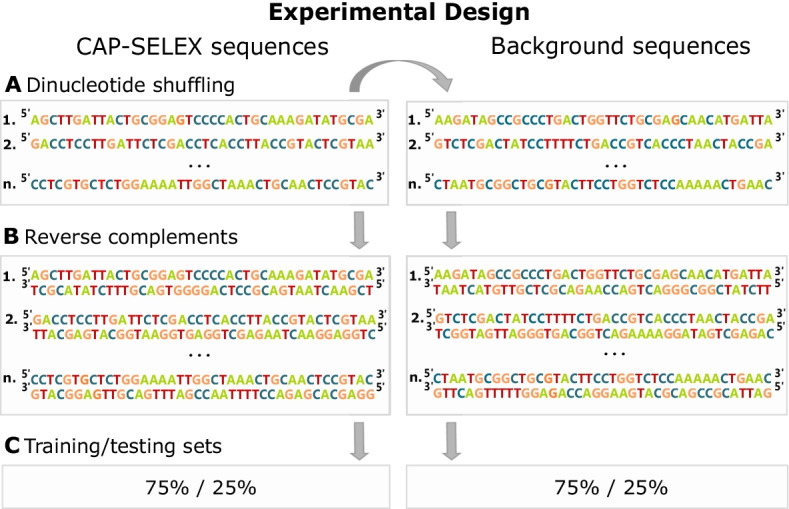
**A** Negative sequences are constructed by shuffling CAP-SELEX sequences with dinucleotide count preservation. **B** We include reverse complements. **C** 75% of DNA double-strands are randomly sampled to training set

We conduct experiments with the 362 CAP-SELEX data sets from [11]. The material comprise 315 unique TF pairings, hence we conduct several experiments for some pairs. A CAP-SELEX data set contains DNA sequences (40 nucleic acids each) purified for containing a binding site of a TF pair specified for that experiment [11]. Therefore, background sets must be constructed artificially. We perform this by shuffling—with dinucleotide count preservation—each CAP-SELEX sequence once with uShuffle tool [25], therefore resulting in equally sized positive and negative sets. Importantly, we consider sequences with their reverse complements. We sample 75% of both positive and negative sequences randomly to a training set with balanced class labels. Hence, 25% of the data is kept as an independent test set (Fig. 1). Furthermore, we sample, with balanced classes, 25% of the training sequences to *pre-training*, where RF parameters are optimised via grid search. In the independent test set, we compare RF model performances with area under receiver-operating characteristic curves (AUROC), and evaluate overall RF model performances in comparison to PWMs trained in [11] with median AUROCs across the experiments (n=362).

### ComBind and JointRF

We model pairwise binding specificities with two RF models: JointRF and ComBind. JointRF utilizes the previously proposed TF-TF pairwise PWMs from [11], while ComBind is a novel approach able to capture multiple pairwise binding configurations simultaneously. Since the exact TF-TF binding site position on a CAP-SELEX sequence or its complement is unknown, we select shorter sub-sequences for RF training with PWMs. JointRF exploits pairwise PWMs from [11] for selecting the most likely binding sites, and trains a RF with them by considering nucleic acids, ordered according to position, as variables for the RF. When scoring an unseen DNA double-strand, JointRF scores all sub-sequences with the same length as variables in RF, and considers the maximum per-position score as the sequence score. Whenever there were multiple motifs reported for a pair in [11], we train JointRF models for each and report the best performing model.

**Fig. 2:**
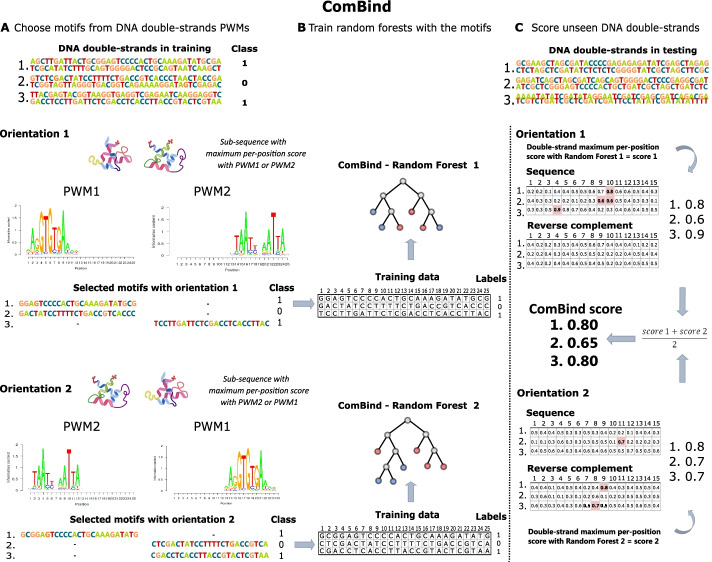
**A** Sub-sequences with maximum PWM scores are chosen from the DNA double-strands according to orientations 1 and 2 (TF1 + TF2 and TF2 + TF1, respectively). PWMs are extended until 25 nucleic acids to cover binding of both TFs. Within an orientation, ComBind scores DNA double-strands with two PWMs and selects the sub-sequence with the highest per-position score. **B** Forests are trained with the chosen sub-sequences separately for both orientations. **C** ComBind scores unseen DNA double-strands with both RFs. Maximum per-position scores are considered as the orientation specific scores, whose average ComBind outputs as the sequence score. [PWM visualization [38], and PWM structures [7].]

ComBind selects the most probable binding sites for RF training with individual TF PWMs (Algorithm 1, Fig. 2). Aim is to select the most probable binding site of one of the TFs, possibly the one with a higher binding specificity, while simultaneously covering binding of the other TF. Thus, prior knowledge regarding the exact TF-TF configuration is not needed. There are four possible binding orientations, which ComBind approximates with two due to reflection of a binding site onto the opposite strand. ComBind trains two random forests, one for each of the orientations: TF1-TF2 and TF2-TF1. Sub-sequences for training the RFs are chosen from DNA double-strands as sub-sequences with maximum PWM scores, similarly as in JointRF. However, ComBind utilizes individual TF PWMs, which are extended by $(0.25, 0.25, 0.25, 0.25)^T$ columns until 25 nucleic acids long—thus including space for the pair without specifying the amount of nucleic acid gaps or overlap between their motifs. In more detail, we extend the PWM with columns, which consider all DNA bases to have the same importance. Thus, DNA in the other TF binding site is not determined in training sequence pre-search. Sub-sequences in TF1-TF2 orientation are selected with TF1 PWM extended to right or TF2 PWM extended to left, while the opposite is true for TF2-TF1 orientation. The selected sub-sequence is always the sub-sequence with maximum score according to either one of the orientation’s two PWMs. Finally, ComBind trains random forests with the chosen 25 nucleic acids long DNA sequences, RF1 for TF1-TF2 orientation and RF2 for TF2-TF1 orientation. Due to PWM elongation to only 25 nucleic acids, even shorter binding sites in the middle of the 40 nucleic acids long CAP-SELEX double-strands can be chosen. ComBind scores unseen DNA double-strands by scoring all 25 nucleic acids long positions with RF1 and RF2, and considers their maximum per-position scores as the corresponding orientation specific scores. ComBind score for a DNA double-strand is then the average of these two scores. Of note, we inspected a couple of pairs and found that averaging outperformed the maximum; possibly due to model stability gained through averaging as both scores do entail information regarding binding affinity. Importantly, the final ComBind score describes TF-TF-DNA binding affinity by considering all possible binding orientations, and can therefore be utilized to give one DNA-binding probability score for a sequence.
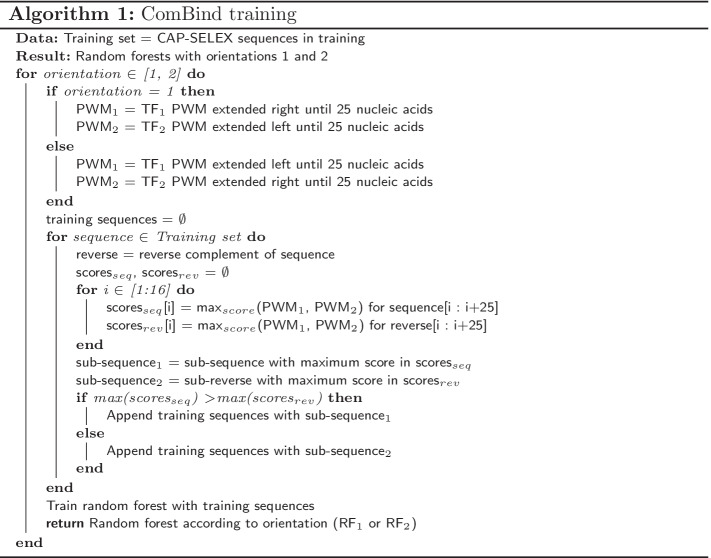


### ComBind outperforms PWM scoring

**Fig. 3:**
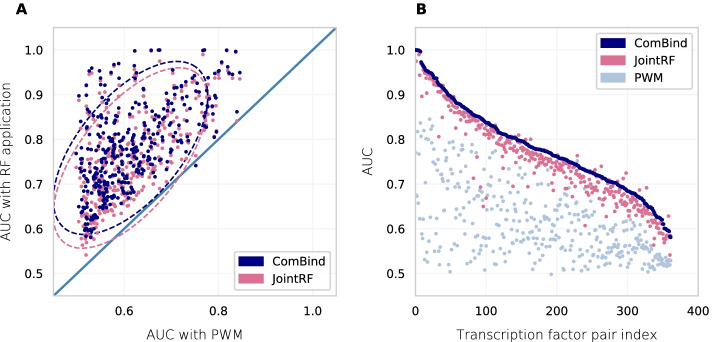
**A** Experimental AUROC relation of ComBind and JointRF to PWM scoring with 2 standard deviation ellipse (N = 362). **B** Summary of experiment AUROCs between the models ordered according to ComBind AUROCs

We score DNA double-strands in 362 independent test sets with corresponding ComBind, JointRF and PWM models; and assess their performance with AUROC values (Additional file 2). JointRF (median AUROC = 0.749) outperforms PWM scoring (0.594, *p*-value < 0.0001, n = 362) (Fig. 3). PWM scoring is described in more detail in the Methods section. Most importantly, when multiple PWMs are provided for a pair from an experiment in [11], we score test set DNA double-strands with each and consider the maximum score as the final PWM score. It is important to note that we compare random forest performance against a defined set of PWMs, not PWM modeling in general. JointRF can be seen as adding a layer of complexity to the already proposed pairwise binding configurations by computing a non-linear prediction for a pairwise binding site with RF. We therefore suggest that RF is a viable option for improving accuracy of orientation and spacing specific TF-TF-DNA binding specificity models, although the eligibility of JointRF in practice should be validated with more comprehensive analyses.

ComBind (median AUROC = 0.775) outperforms PWM scoring (0.594, *p*-value < 0.0001, n = 362), and the difference is slightly greater than for JointRF. In fact, ComBind predicts TF-TF binding in a significantly higher accuracy than JointRF (*p*-value = 0.00195, n = 362). Notice that JointRF exploits PWMs from [11], which were trained with the same CAP-SELEX data that we use, and may therefore give too optimistic results. However, a possible error source in JointRF lies in the noisier random forest training data for pairs with multiple suggested binding configurations (i.e. provided PWMs). JointRF selects sub-sequences for RF training also from positive sequences, which contain a binding site according to another TF-TF spacing, as all training sequences are utilized for training of each configuration specific JointRF model. However, as an ensemble method, random forest should not be extremely sensitive to noise [20]. When scoring sequences with JointRF, all unseen DNA double-strands are scored with the same orientation specific RF, while some sequences may have been bound according to another TF-TF configuration. Of note, we attempted to assign the highest TF pair JointRF score for each test set DNA double-strand separately. However, scoring all sequences with the best-performing JointRF model outperformed slightly the above scoring scheme (AUROC = 0.747, *p*-value = 0.74). Importantly, ComBind (AUROC$_{N=1}$ = 0.775) outperforms JointRF (AUROC$_{N=1}$ = 0.742, *p*-value = 0.00138, n = 203) also for pairs with only one suggested binding configuration (Table 1). ComBind utilizes PWMs derived from completely independent data and outperforms JointRF. Since ComBind considers all gaps at once, binding site prediction accuracy may be increased at least for pairs with more relaxed gap spacing. In fact, it has been suggested that there are TF pairs, which bind DNA rather promiscuously [11].

**Table 1:** Model performances

	Model	Median AUROC
1.	PWM	0.594
2.	JointRF	0.749
3.	ComBind	0.775
4.	JointRF with best configuration score separately for each test sequence	0.747
5.	JointRF for pairs with one configuration	0.742
6.	ComBind for pairs with one configuration	0.775

### Comparing ComBind to support vector machines

We compare ComBind performance to the TF-TF dimer SVM application [19]. We train ComBind models with varying number of sequencing reads using a randomly selected set of five pairs (Additional file 1: Table S1). SVM does not converge properly with large training set size [19], thus, we train SVM models only with 10,000 DNA sequences to which we then compare ComBind performance. Of note, testing is performed with 10,000 unseen DNA sequences. With small training data (N = 10,000); SVM (mean AUROC = 0.744) clearly outperforms ComBind (mean AUROC = 0.694). However, with ComBind, we are able to increase training set size and can show that its performance steadily increases with increasing training set size, until it performs slightly better than SVM with 80,000 training sequences (mean AUROC 0.748).

### ComBind performance vary across TF pairs

**Fig. 4:**
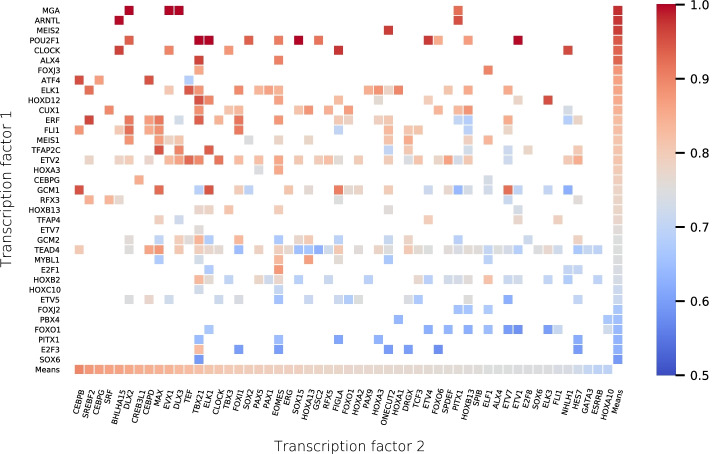
ComBind binding site prediction accuracy (i.e. AUROC) across the studied TF pairs (N = 362). Transcription factors 1 and 2 in the pair are ordered according to their mean ComBind accuracy. Whenever, there are multiple CAP-SELEX libraries for one pair, we consider the one with the highest AUROC

ComBind prediction accuracy varies across TF pairs (Fig. 4). High prediction accuracy may suggest an easier learning problem due to more specific binding preferences or for instance due to a larger- or otherwise purer CAP-SELEX data. Of note, our results suggest that training set size does not greatly affect ComBind prediction accuracy (Additional file 1: Figure S1). In our experiments, binding site prediction problem was easier for some of the pairs. In most cases, ComBind predicts binding with a high accuracy, if the pair includes even one TF with a high mean accuracy across all its ComBind models. For instance, pairs including either POU2F1 (AUROC$_{N=4}$ = 0.980) or MGA (AUROC$_{N=15}$ = 0.935) can be modeled with ComBind with a very high accuracy, while others such as FOXO1 (AUROC$_{N=16}$ = 0.680) and E2F3 (AUROC$_{N=7}$ = 0.629) exhibit lower prediction accuracies in almost all pairwise binding scenarios modeled here. There are also TFs, such as TBX21, for which ComBind accuracy varies greatly depending on the TF it is cooperating with. Furthermore, occasionally TFs loose their high overall ComBind prediction accuracy in a dimeric form with a certain TF (e.g. TEF-ATF4), or bind DNA with a very high DNA binding specificity only when modeled together such as GCM1-ETV7. The examples discussed were selected due to extreme prediction accuracy behaviour and at least four experimental replicates.

### Random forest parameters in ComBind

**Fig. 5:**
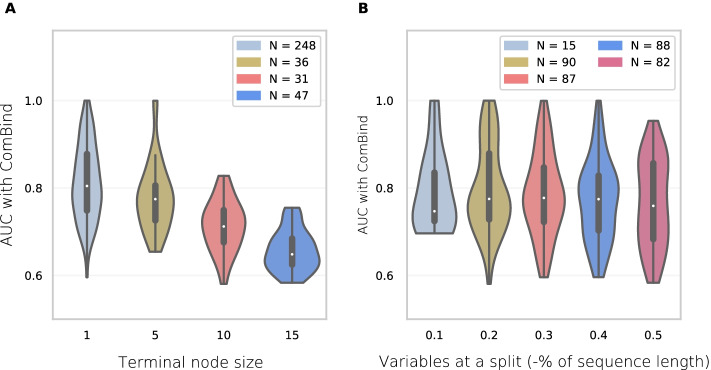
Violin plots of AUROC measures for different random forest parameters in ComBind. The number of TF pairs (N), which obtained the best AUROC with the corresponding parameter value (shown on x-axis) and are included into each violin plot, are represented within the labels. **A** Terminal node size. **B** Number of variables in decision tree data partitioning (as percentage of the amount of total variables)

ComBind and JointRF select a minimum tree node size (1, 5, 10 or 15 instances) and a number of variables randomly assigned to a split function at tree nodes (10%, 20%, 30%, 40% or 50% of the total number of variables) via grid search in *pre-training*. All RFs entail 200 decision trees, due to a good trade-off between model performance and computational cost (Additional file 1: Figure S2). Most of the TF pairs favor small terminal nodes sizes in ComBind (Fig. 5). A high tree depth suggest an easier learning problem and potentially also a more specific DNA binding preference. In fact, ComBind had selected smaller minimum tree node sizes for pairs with higher test set binding site prediction accuracies (Fig. 5).

Greater amount of variables considered at a tree partitioning decrease randomness in a forest and increase variance, which may decrease prediction accuracy. However, the amount of variables should be great enough to correctly capture binding preferences. ComBind had selected values for the ’variables at a split’ parameter rather evenly across the pairs, except that the smallest option (i.e. 10% of sequence length) was chosen only for few pairs (Fig. 5). In ComBind, TF pairs most commonly favor five (20% of 25 nucleic acids sequence length) as the number of variables assigned to split function. Pairs with long binding sites and high binding specificities may prefer smaller percentages. Whenever there are less variables which do not relate to binding with high importance, RF may better exploit the benefits of added randomness with fewer variables sampled for split function at tree nodes.

### ComBind validation with external data

We attempted external validation with data other than CAP-SELEX. Of note, for pairwise TF-DNA binding, experimental methods developed are few. However, an experimental method i.e. SMiLE-seq, which enables pairwise TF-TF-DNA binding site modeling, was developed in [26]. We selected four pairs for which we could find overlapping data between CAP-SELEX and SMiLE-seq (ARNTL-CLOCK, cJUN-cFOS, NR4A2-RXRa, RARa-RXRa), trained ComBind models within CAP-SELEX and tested model performance in the corresponding SMiLE-seq data set (Additional file 1: Table S2). Importantly, these TF pairs were not used for PWM construction in [11], thus, either considered poor-quality data or otherwise not exhibit pairwise TF-DNA binding. ComBind models are likely not trained here with optimal data. Of note, DNA read length is 101 base pairs in SMiLE-Seq, while we optimised the models for 40 base pairs. Interestingly, ComBind still performs rather well on SMiLE-seq (mean AUROC = 0.625). Performance vary greatly depending on the TF pair; ComBind predicts ARNTL-CLOCK binding well (AUROC = 0.784), while for the other three pairs prediction accuracy is not as high. When comparing to PWMs developed in [26], PWMs outperform ComBind, although the result slightly depends upon the PWM scoring scheme. If we assign the highest PWM score for each DNA sequence when multiple PWMs are provided, as usually done in practice, ComBind performs closer to PWM (mean AUROC = 0.660). However, one PWM predicts cJUN-cFOS binding very accurately, thus, if we use only the best-performing PWM, the difference is greater (mean AUROC=0.669). It must be noted that although ComBind performs reasonably well within this external validation, more research is needed to accurately describe its performance in relation to PWMs due to limited sample size and unknown reliability of sequence libraries utilized for ComBind training.

In addition, we evaluated ComBind performance in three experimental replicates [11], i.e. models are trained in different CAP-SELEX experiments than within which they are evaluated. We selected three pairs randomly. ComBind performs well on these experimental replicates (mean AUROC = 0.740) and outperforms PWMs (mean AUROC = 0.644, Additional file 1: Table S2). Although ComBind models are here trained with CAP-SELEX libraries utilized also in [11], making the models more reliable, sample size is small and definite conclusions cannot be drawn.

### Binding specificity visualization

ComBind and JointRF binding specificity models cannot be directly visualized. We therefore construct PWMs from positive RF out-of-bag prediction sequences for two randomly selected pairs: ALX4-EOMES and ERF-MAX (Additional file 1: Figures S3 and S4). Relaxed gap spacings and orientation unspecificity in ComBind prevent informative binding specificity visualizations. Thus, we applied visualization with JointRF, where decision tree variables are the nucleic acids ordered as positioned on a configuration specific binding site from [11]. JointRF decreases information content of nucleic acids from the PWMs. The most important nucleic acids regarding binding specificity remain unchanged, and only small changes can be observed in nucleic acid importance relations within the binding sites. JointRF captures also higher-order dependencies, which may decrease PWM information content of individual nucleic acids.

## Discussion

Predicting TF-DNA binding is a key for understanding gene expression patterns. Defining TF-DNA interactions has however proved challenging, not only due to the complexity of DNA binding motifs, but due to the varying manners TFs function. Transcription factors cooperate with each other, interact with nucleosomes and recruit different cofactors involved in gene expression networks [1]. Despite the high importance of TF cooperativity in gene expression, research has not yet been extensive. According to current knowledge, TFs may cooperate directly by forming TF-TF complexes either in a manner independent of DNA, facilitated by DNA or even entirely mediated by DNA [9]. In [11], Jolma et al. demonstrated DNA binding for 315 TF pairs with CAP-SELEX; a SELEX-seq based method for pairwise binding, and identified in total 618 distinct DNA binding motifs. With these data, we aimed to improve prediction accuracy of current pairwise TF-TF-DNA binding models with an approach that includes multiple putative TF-TF binding configurations in one binding score.

TF-DNA binding specificites are commonly modeled with PWMs. Despite the PWM being an intuitive motif description, it may be too simple and inflexible for an accurate binding site prediction. Limitations include the independent normalization of positions during PWM construction and the assumed independence of nucleic acids on the motif [16], which may not always hold [13, 15]. Of note, motif position dependencies may not be restricted to nearest neighbors [15]. The PWM might be especially unsuitable for cooperative binding due to the varying binding orientations and number of nucleic acid gaps or overlap between the motifs. We demonstrated how RF based models outperform the PWM in TF-TF-DNA binding site prediction within CAP-SELEX. JointRF simply adds a layer of complexity with RF to the already proposed TF-TF configuration specific joint PWMs [11], by selecting sub-sequences for RF training with them and by utilizing the sub-sequence nucleic acids as features for decision trees. When scoring unseen DNA, JointRF significantly outperformed PWM scoring. However, one limitation in the present study is that we compared RF performance to a limited selection of PWMs. RF can achieve high modeling complexity by assembling decision trees, while maintaining model stability with bootstrap aggregation and random variable selection at tree nodes [20]. Our results demonstrate that increasing mathematical complexity of TF pair motif descriptions improves prediction accuracy on unseen DNA.

We proposed a flexible RF based model, ComBind, for pairwise DNA binding without fixed binding configurations. Thus, an unseen DNA double-strand may be given a TF-TF binding site score without pre-defining the pair’s orientation. ComBind trains a RF for both putative orientations: TF1-TF2 and TF2-TF1. Of note, both orientations include the possibility for opposite strand binding. ComBind selects sub-sequences for RF training with individual TF PWMs extended with $(0.25, 0.25, 0.25, 0.25)^T$ columns until 25 nucleic acids—either left or right according to the considered orientation—thus covering binding space of the other TF. RFs exploit nucleic acids on the selected sub-sequences as features. We attempted to maintain appropriate binding site length with the defined 25 nucleic acids, while allowing sub-sequence selection from the middle of training sequences. Of note, it has been discussed how DNA binding models benefit from extended binding sites with ’flanking regions’ [15]. In the independent test set, ComBind significantly outperformed PWM scoring and JointRF; even though JointRF may be prone to overfitting as it exploits pairwise PWMs trained previously with the same CAP-SELEX data [11]. Of note, we found that the Hong et al. pairwise SVM model outperformed ComBind with small training data [19], but we demonstrated improving prediction accuracies with ComBind with respect to increasing training set size. Thus, ComBind is a viable option whenever high-throughput data is available. ComBind may yield higher prediction accuracies especially for pairs with flexible binding preferences due to its capability to model binding without defined configurations, even though sub-sequences selected for RF training have thus higher disparity in ComBind than in JointRF, complicating the learning task. Decision trees have the capability to identify clusters of correlated features, while trees in RF select variables randomly at each node for the split function to consider [20, 21], thus, enabling the discovery of groups of data with correlated variables even at altered positions within training sequences. Finally, ComBind scores an unseen DNA double-strand with both RFs and selects maximum per-position score as the sequence’s orientation specific RF score (TF1-TF2 or TF2-TF1). ComBind defines an average of these two scores as the final score. Averaging may increase model stability. Whenever TFs are bound to complementary strands, both RFs should be capturing the same binding preference. Even when a pair exhibit only same strand binding, both orientation specific RFs should include evidence for at least one TF. Finally, we found ComBind to predict TF-TF-DNA binding reasonably well also within external validation using SMiLE-seq data and CAP-SELEX experimental replicates [11, 26]. However, PWM outperformed ComBind with SMiLE-seq. ComBind training data in SMiLE-seq validation was likely sub-optimal (i.e. CAP-SELEX libraries not used for PWM construction in [11]), and more research is needed to confirm random forest usability for practical applications. Importantly, external validation sample size performed here was small, which adds uncertainty to the conclusions.

Random forest is able to process large high-throughput genetic data sets. The downside, however, is that binding preferences cannot be readily visualized. Although ComBind can include multiple binding configurations into one prediction, it cannot be utilized for research on the characteristics of pairwise binding specificities e.g. to determine motif spacings and orientation. For instance, the SVM model proposed in [19], enable more specific binding preference examination, although being more suitable for smaller data sets. However, we did examine the degree of ComBind prediction accuracy across TFs, which suggested an easier learning problem for pairs entailing at least one TF with high overall prediction accuracy. Of note, some TFs showcased high pairwise binding prediction accuracies only when modeled together, and vice versa. One limitation of this study is that random forests trained with high-throughput genetic data are large and storing them requires substantial computational memory. In addition, external validation performed here for ComBind was limited due to small sample size and unknown quality of training data. More research is needed to validate ComBind performance—especially in experimental data other than CAP-SELEX—to ensure good prediction accuracies for practical applications. We demonstrated that random forest is suitable for modeling TF pair DNA binding preferences, and our results suggest that TF pairs benefit from binding specificity models with configuration flexibly.

## Conclusions

We developed random forest based TF-TF-DNA binding specificity models: JointRF and ComBind, which both significantly outperformed PWM scoring. Models were trained and their performances were assessed with 362 CAP-SELEX sequence libraries from [11]. We utilized previously proposed pairwise PWMs in JointRF and demonstrated that adding complexity to motif representation with random forest improves binding site prediction accuracy. ComBind is a flexible random forest based model for pairwise DNA binding able to score DNA sequences with high accuracy without defining binding configurations. ComBind outperforms PWM scoring as well as JointRF. However, in external validation the results are more uncertain. It is important to note that we compared ComBind performance only to a defined set of PWMs and mainly within CAP-SELEX sequence libraries. Thus, more experimental and computational research is needed to verify good prediction accuracies with ComBind before utilizing it in practical applications. In conclusion, we demonstrate that random forest is well suited for modeling TF pair DNA binding specifities, and propose a model, ComBind, able to predict binding sites without previous knowledge on pairwise binding configurations.

## Methods

### Materials

CAP-SELEX is a method enabling the discovery of TF-TF-DNA binding preferences—also when facilitated by DNA [11]. In short, DNA double-strands are mixed with two differently tagged TFs, and purified for pairwise binding, after which they are PCR amplified and sequenced [11]. This is repeated three times. The resulting CAP-SELEX library contains hundreds of thousands of DNA sequences known to entail a binding site; either on the sequence itself or on its complement. However, the length and exact position of the motif on the DNA double-strand is unknown. In [11], Jolma et al. tested 9400 TF pairs for pairwise binding: 315 bound DNA cooperatively with novel motifs. In total, they derived 618 pairwise PWMs from 362 CAP-SELEX data sets. We performed experiments with the 362 CAP-SELEX libraries, thus including multiple experiments for some pairs. CAP-SELEX libraries are available in [27]. JointRF exploits the 618 pairwise PWMs in training, while ComBind uses PWMs derived for the TFs individually from high-throughput SELEX (HT-SELEX) experiments in [7, 11]. Although, PBX4 PWM was available only from UniProbe data base [28, 29].

We constructed the negative set for classification artificially by shuffling, with dinucleotide count preservation, each CAP-SELEX DNA sequence once with uShuffle tool [25]. Of note, dinucleotides appear hierarchically in DNA [30]—aim is to model differences between specific TF binding sites rather than differences between binding sites in general and other regions of DNA, hence the dinucleotide preservation.

We performed external validation with SMiLE-seq data [26]. SMiLE-seq data sets comprise 101 base pair long DNA sequences for which we similarly built the negative set with dinucleotide count preservation [25]. Of note, we trained RFs with the entire CAP-SELEX data for SMiLE-seq validation with the smallest data sets (NR4A2-RXRa, RARa-RXRa and JUN-FOS), e.g. NR4A2-RXRa data contain still only 17,804 sequencing reads in RF training. We compared ComBind performance to joint pairwise PWMs [26]. Of note, PWMs are likely trained within the same data sets than they are here evaluated in.

### Random forest

Random forest assembles decision trees (in here classification trees) [20], which recursively divide the data [31]. At each node, a split function selects a split feature and its label by minimizing cost function, which in here is the Gini-index, $\sum _{i=1} {\hat{p}}_i(1-{\hat{p}}_i)$, where ${\hat{p}}_i$ is the proportion of instances belonging to class *i* [32, 33]. We decide whether an impure node is split further with a minimum node size. ComBind and JointRF select the optimal minimum tree node size in pre-training (N = {1, 5, 10 or 15}). Near purity trees have been suggested to perform better with genomic data due the bias-lowering effect of deep trees in the presence of high feature space, although in general, larger data sets may require larger terminal node sizes [21, 34].

Decision trees are unstable and the algorithms may stagnate at local optima. Random forest addresses these issues by combining bootstrap aggregation (i.e. bagging) and random variable selection at tree nodes [20]. Instances in training data are randomly with replacement divided into subsections for training classification trees, which are combined in the final classification decision [20, 35, 36]. Thus, part of the decision trees have been trained without outliers and will have more power in the aggregation decision. In this study, one third of training instances are assigned to an out-of-bag sample when learning a tree. Furthermore, we consider the number of decision trees as a hyperparameter, and therefore evaluated ComBind performance with eight TF pairs and with varying number of trees in RF (N={25, 50, 75, 100, 125, 150, 200 and 250}). Random forests in ComBind and JointRF comprise 200 classification trees due to a good trade-off between computational cost and model performance (Additional file 1: Figure S2). Random selection of features for split function at tree nodes further decorrelates the decision trees [20]. ComBind and JointRF select the optimal number of variables, as percentage from total number of variables ({10%, 20%, 30%, 40% and 50%} rounded in R software, thus following EC 60559 standard), with grid search along with minimum node size within pre-training.

Nucleic acids on the most probable binding site are utilized as RF features. Variables are the nucleic acids $N_i \in$ {A, C, G, T} ordered according to their position, $i = (1,..,n)$, on the most probable binding site, ${\mathbf {x}} = (N_1,...,N_{n})$. We implement binding specificity models in R software with randomForest package [37].

### PWM scoring

When scoring sequences only with pairwise PWMs, DNA sequences are padded with five ’N’ variables, scored with a probability of 0.25 (i.e. zero in log-odds scoring), at the ends due to improved prediction accuracy (Additional file 1: Figure S5). PWM score in final random forest to PWM comparison is the maximum per-position log-odds score, although in ComBind and JointRF sub-sequence selection we had utilized PWMs as position probability matrices. Of note, we replaced $-\,\infty$ PWM scores, if still present after maximum per-position score selection, with the data set minimum PWM score. Finally, whenever multiple PWMs were reported for a pair from a CAP-SELEX experiment [11], we score DNA double-strands with all and assign the highest score for each DNA double-strand.

### Model comparisons

We train models with 75% of the data and test performance in the remaining 25%. However, there are two CAP-SELEX libraries with an especially high number of sequences (i.e. TFAP4-ETV1 and TFAP4-ETV4), for which we train RFs with a smaller amount of data. Hence we sample only 50% of sequences for training and utilize the remaining 50% in testing of these models. We compare three models to each other: ComBind, JointRF and PWM scoring. Of note, we assess performances of the models within a few additional modeling schemes. Statistical significance are inferred with Wilcoxon signed-rank test. Finally, we conducted a ComBind performance comparison across the studied TFs, in which we present for each pair the highest AUROC value obtained from its experiments, whenever PWMs were derived from multiple CAP-SELEX libraries for the TF-TF pair in [11].

## Supplementary Information

**Additional file 1.** Supplemental Tables and Figures.

**Additional file 2.** Excel sheet of TF pair AUROC values with studied CAP-SELEX libraries (N = 362).

## Data Availability

R software implementation of ComBind is available in GitHub: https://github.com/AnniAleksandra/ComBind The CAP-SELEX data sets, provided in [11], and analysed during the current study are available in the European Nucleotide Archive (ENA), Project: PRJEB7934, https://www.ebi.ac.uk/ena/browser/view/PRJEB7934 [27]

## Abbreviations

TF: Transcription factor
PWM: Position weight matrix
RF: Random forest
AUROC: Area under receiver operating characteristics curve
CAP-SELEX: Consecutive affinity purification systematic evolution of ligands by exponential enrichment

## References

[1] Lambert SA, Jolma A, Campitelli LF, Das PK, Yin Y, Albu M (2018). The human transcription factors. Cell.

[2] Stormo GD, Zhao Y (2010). Determining the specificity of protein-DNA interactions. Nat Rev Genet.

[3] Rohs R, West SM, Sosinsky A, Liu P, Mann RS, Honig B (2009). The role of DNA shape in protein-DNA recognition. Nature.

[4] Zhou T, Shen N, Yang L, Abe N, Horton J, Mann RS (2015). Quantitative modeling of transcription factor binding specificities using DNA shape. Proc Natl Acad Sci.

[5] Yin Y, Morgunova E, Jolma A, Kaasinen E, Sahu B, Khund-Sayeed S (2017). Impact of cytosine methylation on DNA binding specificities of human transcription factors. Science.

[6] Badis G, Berger MF, Philippakis AA, Talukder S, Gehrke AR, Jaeger SA (2009). Diversity and complexity in DNA recognition by transcription factors. Science.

[7] Jolma A, Yan J, Whitington T, Toivonen J, Nitta KR, Rastas P (2013). DNA-binding specificities of human transcription factors. Cell.

[8] Mayran A, Sochodolsky K, Khetchoumian K, Harris J, Gauthier Y, Bemmo A (2019). Pioneer and nonpioneer factor cooperation drives lineage specific chromatin opening. Nat Commun.

[9] Morgunova E, Taipale J (2017). Structural perspective of cooperative transcription factor binding. Curr Opin Struct Biol.

[10] Wunderlich Z, Mirny LA (2009). Different gene regulation strategies revealed by analysis of binding motifs. Trends Genet.

[11] Jolma A, Yin Y, Nitta KR, Dave K, Popov A, Taipale M (2015). DNA-dependent formation of transcription factor pairs alters their binding specificity. Nature.

[12] Jolma A, Kivioja T, Toivonen J, Cheng L, Wei G, Enge M (2010). Multiplexed massively parallel SELEX for characterization of human transcription factor binding specificities. Genome Res.

[13] Bulyk ML, Johnson PL, Church GM (2002). Nucleotides of transcription factor binding sites exert interdependent effects on the binding affinities of transcription factors. Nucleic Acids Res.

[14] Bailey TL, Elkan C. The value of prior knowledge in discovering motifs with MEME. In: Ismb. 1995;3:21–29. 7584439

[15] Siddharthan R (2010). Dinucleotide weight matrices for predicting transcription factor binding sites: generalizing the position weight matrix. PLoS One.

[16] Ruan S, Stormo GD (2017). Inherent limitations of probabilistic models for protein-DNA binding specificity. PLoS Comput Biol.

[17] Guo Y, Tian K, Zeng H, Guo X, Gifford DK (2018). A novel k-mer set memory (KSM) motif representation improves regulatory variant prediction. Genome Res.

[18] Alipanahi B, Delong A, Weirauch MT, Frey BJ (2015). Predicting the sequence specificities of DNA-and RNA-binding proteins by deep learning. Nat Biotechnol.

[19] Hong C, Yip KY (2020). Flexible k-mers with variable-length indels for identifying binding sequences of protein dimers. Brief Bioinform.

[20] Breiman L (2001). Random forests. Mach Learn.

[21] Chen X, Ishwaran H (2012). Random forests for genomic data analysis. Genomics.

[22] Touw WG, Bayjanov JR, Overmars L, Backus L, Boekhorst J, Wels M (2013). Data mining in the Life Sciences with Random Forest: a walk in the park or lost in the jungle?. Brief Bioinform.

[23] Wang X, Lin P, Ho JW (2018). Discovery of cell-type specific DNA motif grammar in cis-regulatory elements using random Forest. BMC Genomics.

[24] Ardakani FB, Schmidt F, Schulz MH (2018). Predicting transcription factor binding using ensemble random forest models. F1000Research.

[25] Jiang M, Anderson J, Gillespie J, Mayne M (2008). uShuffle: a useful tool for shuffling biological sequences while preserving the k-let counts. BMC Bioinform.

[26] Isakova A, Groux R, Imbeault M, Rainer P, Alpern D, Dainese R (2017). SMiLE-seq identifies binding motifs of single and dimeric transcription factors. Nat Methods.

[27] European Nucleotide Archive (ENA) Analysis of heterodimeric transcription factor complex specificities; 2015. Project: PRJEB7934. https://www.ebi.ac.uk/ena/browser/view/PRJEB7934.

[28] UniProbe data base: PBX4; 2016. Accession number: UP00613. http://thebrain.bwh.harvard.edu/uniprobe/details34.php?id=613.

[29] Barrera LA, Vedenko A, Kurland JV, Rogers JM, Gisselbrecht SS, Rossin EJ (2016). Survey of variation in human transcription factors reveals prevalent DNA binding changes. Science.

[30] Nussinov R (1991). Compositional variations in DNA sequences. Bioinformatics.

[31] Kingsford C, Salzberg SL (2008). What are decision trees?. Nat Biotechnol.

[32] Krzywinski M, Altman N (2017). Points of significance: classification and regression trees.

[33] Breiman L, Friedman J, Stone CJ, Olshen RA (1984). Classification and regression trees.

[34] Lin Y, Jeon Y (2006). Random forests and adaptive nearest neighbors. J Am Stat Assoc.

[35] Breiman L (1996). Bagging predictors. Mach Learn.

[36] Skurichina M, Duin RP (2002). Bagging, boosting and the random subspace method for linear classifiers. Pattern Anal Appl.

[37] Liaw A, Wiener M. Classification and regression by randomForest. R News. 2002;2(3):18–22. https://CRAN.R-project.org/doc/Rnews/.

[38] Bembom O. seqLogo: sequence logos for DNA sequence alignments; R package version 1.40.0. 2016.

