# Supplementary material for: Modeling binding specificities of transcription factor pairs with random forests
Source: BMC Bioinformatics. 2022 Jun 3;23:212. doi: 10.1186/s12859-022-04734-7 (PMC9166390; doi:10.1186/s12859-022-04734-7)
Supplement: Supplementary file 1 — Additional file 1. Supplemental Tables and Figures. [file 12859_2022_4734_MOESM1_ESM.pdf]

Supplementary Material

Modeling binding specificities of transcription factor pairs with random forests

Anni Antikainen[1,2,3*], Markus Heinonen[1,4], Harri Lähdesmäki[1]

1. Department of Computer Science, Aalto University, Espoo, 02150, Finland.
2. Folkhälsan Institute of Genetics, Folkhälsan Research Center, FI-00290, Helsinki, Finland.
3. Research Program for Clinical and Molecular Metabolism, Faculty of Medicine, University of Helsinki, FI-00290, Helsinki, Finland.
4. Helsinki Institute for Information Technology, Finland.

Table of Contents 0
Figure S1 2
Figure S2 3
Figure S3 4
Figure S4 5
Figure S5 6
Table S1 7
Table S2 8
References 9

Figure S1

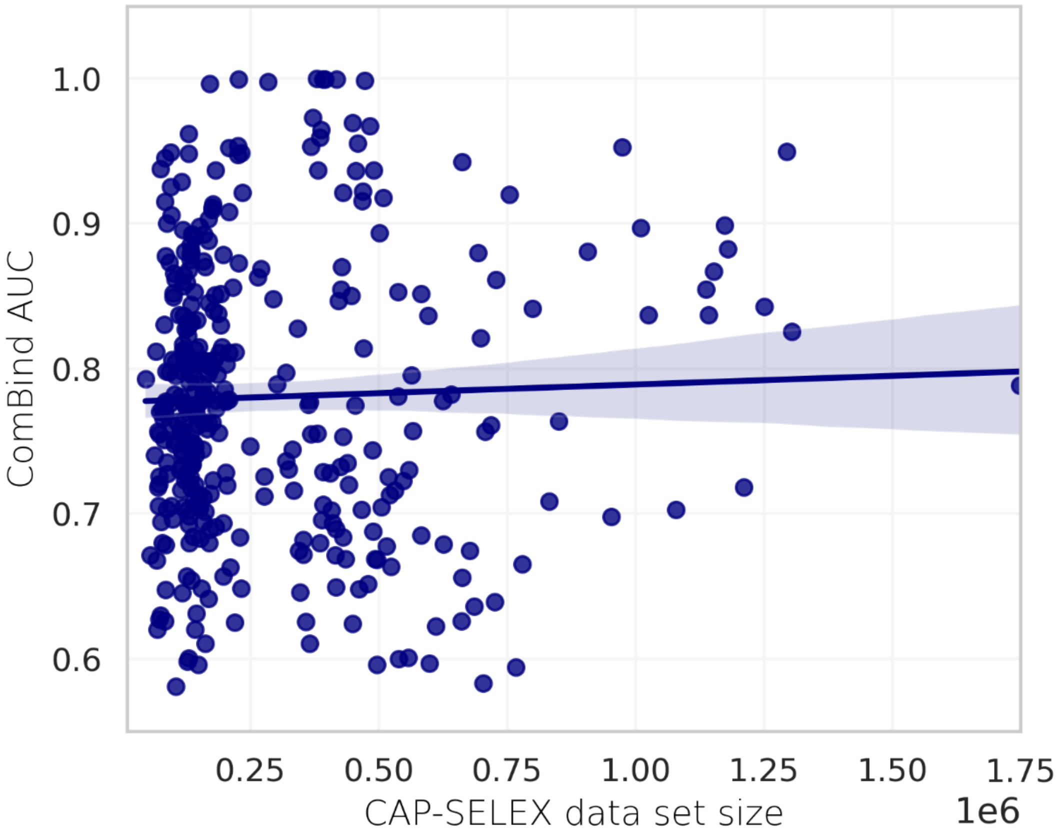

ComBind binding site prediction accuracy (AUC) across all TF pairs (n=362) with respect to CAP-SELEX data set size.

Figure S2

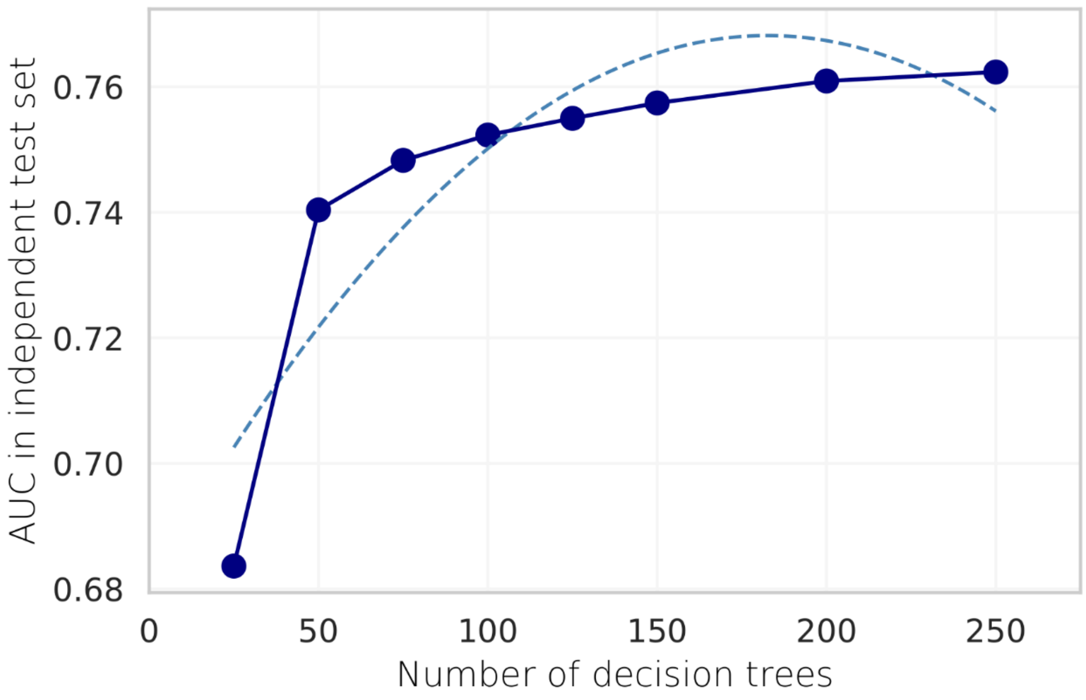

AUC in independent test set with varying number of decision trees in JointRF random forest. Mean of performance with 17 randomly selected TF pairs: ALX4-EOMES, ALX-TBX21, ARNTL-PITX11, ATF4-CEBPB, ATF4-CEBPD, CLOCK-EVX1, ETV2-Tef, ETV5-DRGX, ETV5-EOMES, ETV5-EVX1, GCM2-ONECUT2, GCM2-PITX1, GCM2-TBX21, HOXA3-PAX5, HOXD12-HOXA3, TEAD4-CEBPB, TEAD4-EOMES.

Figure S3

A

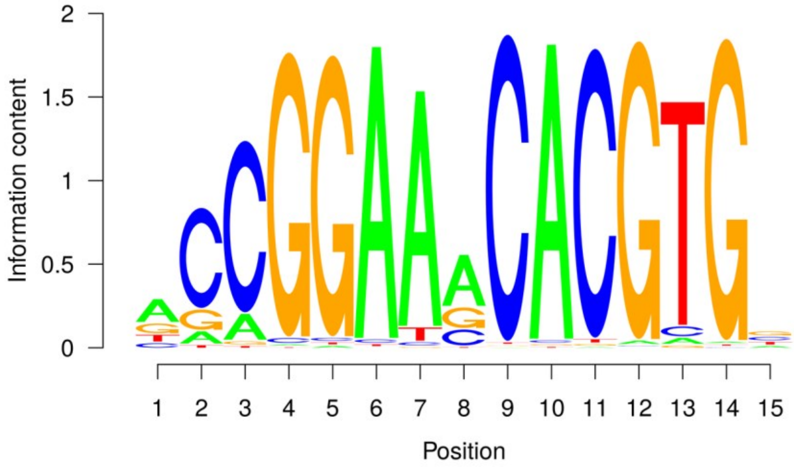

B

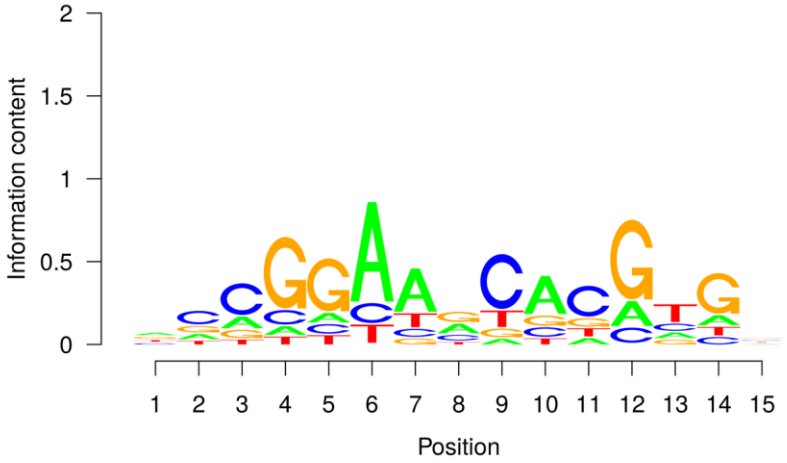

(A) PWM of ERF-MAX from Jolma et al (2015) [1], and **(B)** ERF-MAX PWM constructed from JointRF positive out-of-bag sequences.

Figure S4

A

B

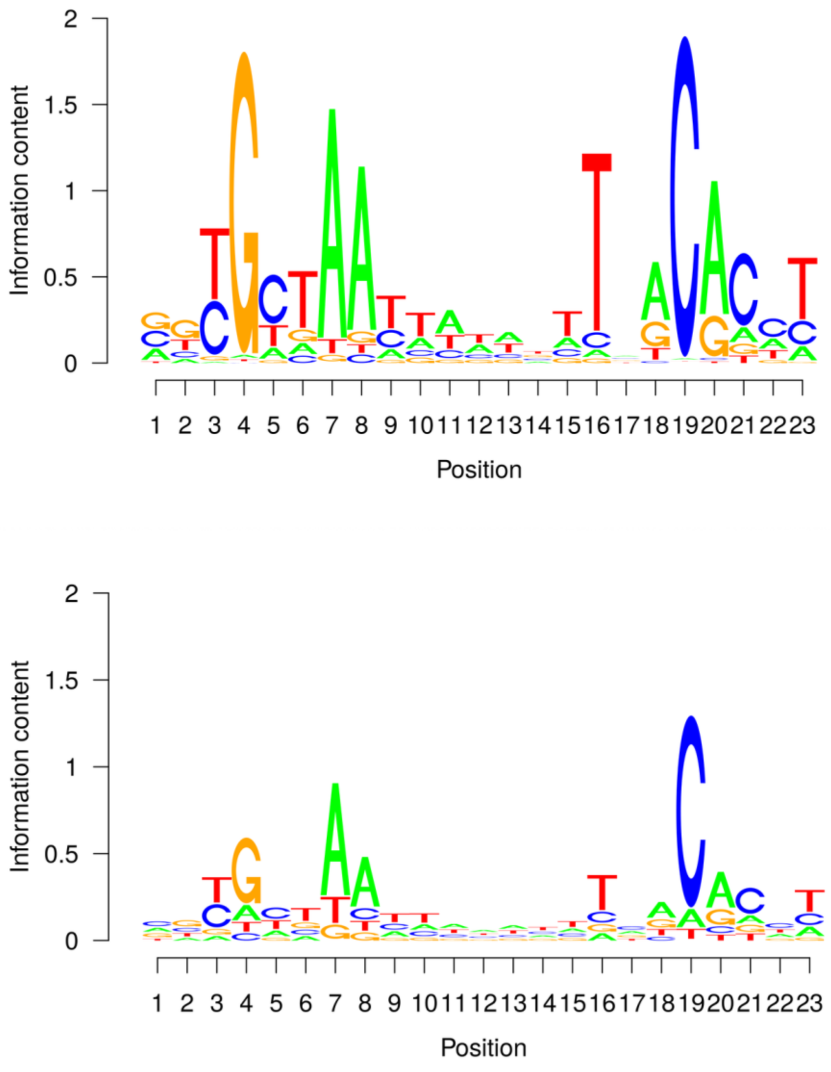

(A) PWM of ALX4-EOMES from Jolma et al (2015) [1], and **(B)** ALX4-EOMES PWM constructed from JointRF positive out-of-bag sequences.

Figure S5

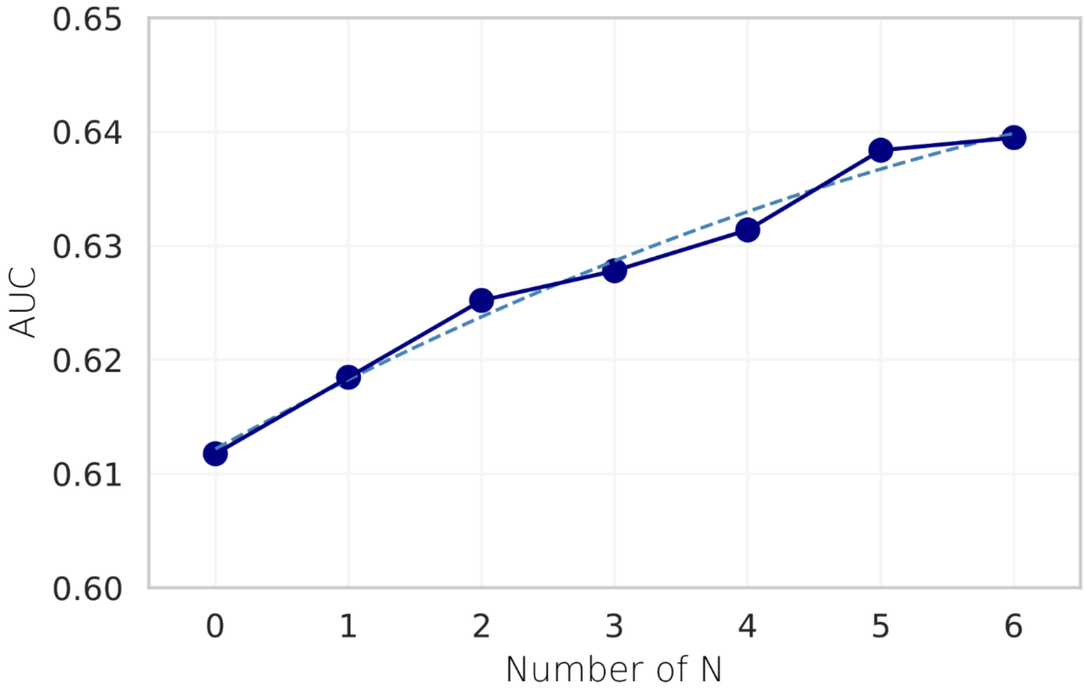

PWM scoring AUC with varying number of N in sequence read padding. PWMs are here utilized as position probability matrices (PPMs) and mean-probability score as the read-specific score. Mean of performance with 25 randomly selected TF pairs (same pairs as in Supplemental Figure 1, in addition to eight further randomly selected pairs): ALX4-EOMES, ALX-TBX2, ARNTL-PITX1, ATF4-CEBPB, ATF4-CEBPD, CLOCK-EVX1, ETV2-TEF, ETV5-DRGX, ETV5-EOMES, ETV5-EVX1, GCM2-ONECUT2, GCM2-PITX1, GCM2-TBX21, HOXA3-PAX5, HOXB2-EOMES, HOXD12-HOXA3, TEAD4-CEBPB, TEAD4-EOMES, TTEAD4-ERG, Erf-FIGLA, ETV2-EVX1, MYBL1-ELF1, MGA-PITX1, ETV5-HOXB13, FLI1-BHLHA15.

Table S1

Comparison to support vector machine (SVM)[2] TF-TF pairwise DNA binding prediction model (Hong et al. (2020)). SVM is trained only with 10,000 DNA sequences (balanced classes) due to improved accuracy. ComBind enables training within a larger training set, and we show that performance improves with increasing training data (N). Comparison is performed with five transcription factor pairs. Testing is always performed in the same 10,000 DNA reads.

	**SVM**	**ComBind**				
**TF-TF pair**	**N=10,000**	**N=10,000**	**N=20,000**	**N=40,000**	**N=50,000**	**N=80,000**
ALX4-EOMES	0.785	0.749	0.780	0.801	0.821	0.836
CUX1-RFX5	0.847	0.799	0.806	0.816	0.819	0.823
E2F3-FOXI1	0.611	0.551	0.566	0.574	0.581	0.582
ERF-FIGLA	0.754	0.700	0.719	0.742	0.750	0.757
RFX3-HES7	0.724	0.671	0.692	0.717	0.734	0.740
**Mean AUROC**	0.744	0.694	0.713	0.730	0.741	0.748

Table S2

ComBind validation in **A.** SMiLE-Seq data[3], and **B.** CAP-SELEX experimental replicate. Experiments in which the RFs are trained (**B**): GCM1_HOXB13_2_AU_TGCGAC40NGGT, POU2F1_ELK1_2_AX_TAGCGA40NGCT, TEAD4_HOXB13_2_AX_TGTGCA40NCCG.

A.

**TF-TF pair**	**ComBind AUROC**	**PWM AUROC**	**Best performing PWM AUROC**
ARNTL-CLOCK	0.7836	0.5871	0.5871
NR4A2-RXRa	0.5938	0.5382	0.5382
RARa-RXRa	0.5461	0.6118	0.6118
JUN-FOS	0.5749	0.9027	0.9401
**Mean AUROC**	0.6246	0.6600	0.6693

B.

**TF-TF pair**	**CAP-SELEX experimental replicate**	**ComBind AUROC in experimental replicate**	**PWM AUROC**
GCM1-HOXB13	GCM1_HOXB13_3_AX_TGCGAC40NGGT	0.7261	0.5122
POU2F1-ELK1	POU2F1_ELK1_2_AS_TGACGA40NGCA	0.7688	0.7669
TEAD4-HOXB13	TEAD4_HOXB13_3_AY_TCTATG40NTAG	0.7259	0.6521
**Mean AUROC**		0.7402	0.6438

References

1. Jolma, A., Yin, Y., Nitta, K. R., Dave, K., Popov, A., Taipale, M., Enge, M.,Kivioja, T., Morgunova, E., and Taipale, J. (2015). DNA-dependent formation of transcription factor pairs alters their binding specificity. Nature, 527(7578), 384
2. Hong C, Yip KY. Flexible k-mers with variable-length indels for identifying binding sequences of protein dimers. Briefings in Bioinformatics. 2020;21(5):1787–1797.
3. Isakova, A., Groux, R., Imbeault, M. *et al.* SMiLE-seq identifies binding motifs of single and dimeric transcription factors. Nat Methods **14,** 316–322 (2017).
